# Structural recurrence in well-differentiated papillary thyroid carcinoma: a 10-year single center cohort study

**DOI:** 10.3389/or.2025.1638499

**Published:** 2025-11-20

**Authors:** Francisco Araújo Dias, Rafael Pereira de Souza, Tabata Briaunys Milan, Rafael De Cicco

**Affiliations:** Head and Neck Department - Instituto de Cancer Dr. Arnaldo, São Paulo, Brazil

**Keywords:** thyroid neoplasms, cancer recurrence, thyroidectomy, extrathyroid extension, papillary thyroid carcinoma, differentiated thyroid cancer, structural recurrence, disease-free survival

## Abstract

**Introduction:**

Well-differentiated papillary thyroid carcinoma (PTC) generally carries an excellent prognosis; however, certain pathological features such as gross extrathyroidal extension and tumor diameter ≥2 cm have been associated with structural disease recurrence. This study aimed to identify the key clinical and histopathological factors associated with disease-free survival in patients undergoing total thyroidectomy for PTC.

**Methods:**

A retrospective cohort study was conducted including 750 patients who underwent total thyroidectomy with or without neck dissection between 2014 and 2024 at a tertiary academic cancer center. Clinical, pathological, and oncological follow-up data were analyzed over a maximum follow-up period of 100 months. Kaplan–Meier survival analysis, log-rank testing, and Cox proportional hazards regression were employed for statistical evaluation.

**Results:**

The structural recurrence rate was 4%, and overall survival reached 99%. In the multivariate analysis, only gross extrathyroidal extension (HR 3.29; p = 0.008) and tumor diameter (HR 1.32; p = 0.013) were independently associated with recurrence. Variables such as age, smoking status, perineural invasion, vascular invasion, and central lymph node involvement did not show significant associations with structural recurrence.

**Conclusion:**

Gross extrathyroidal extension and increased tumor diameter were identified as the primary prognostic factors for structural recurrence in patients with PTC. Multicenter studies are warranted to validate these findings in the broader Brazilian population.

## Introduction

Well-differentiated papillary thyroid carcinoma represents about 84% of all thyroid malignancies. Despite its high incidence and prevalence, the overall survival rate remains around 98.5%, with the majority of cases presenting with tumors smaller than 1 cm in diameter (1). In Brazil, the estimated annual incidence between 2023 and 2025 is 16,660 cases, corresponding to approximately 7.68 cases per 100,000 habitants (2).

The disease-free survival (DFS) rate following surgical treatment is approximately 85.8% at 3 years, with structural recurrence most frequently observed in cervical lymph nodes and lung (3).

Several factors are associated with structural recurrence, including lymph node metastasis, multicentricity, lymphovascular invasion, and extrathyroidal extension (ETE). Among these, extrathyroidal extension has the most significant impact on recurrence (4).

The 2015 American Thyroid Association (ATA) guidelines on differentiated thyroid cancer identify histological subtype, molecular markers, lateral compartment lymph node involvement, and extrathyroidal extension as key predictors of disease recurrence. ETE, in particular, is recognized as a major determinant of poor prognosis (5).

Previous studies have suggested that tumor size greater than 2 cm is associated with reduced disease-free survival when accompanied by additional risk factors, and does not independently influence overall survival or recurrence-free survival (6–8).

Although systemic therapies such as sorafenib, lenvatinib, and cabozantinib have expanded treatment options for advanced thyroid cancer, their indications remain restricted to cases of unresectable tumors, distant metastases, or aggressive histologic variants (5).

Hence, thyroidectomy remains the primary treatment modality for well-differentiated papillary thyroid cancers, offering the highest likelihood of achieving long-term disease control (5).

## Methods

This retrospective cohort study was conducted at the Dr. Arnaldo Cancer Institute (Instituto de Câncer Doutor Arnaldo), a tertiary center in São Paulo, Brazil. Between April 2014 and November 2024, 750 consecutive patients who underwent total thyroidectomy with or without neck dissection for histologically confirmed well-differentiated papillary thyroid carcinoma were included. The postoperative follow-up duration ranged from 6 to 100 months.

The primary outcome of this study was to evaluate factors associated with disease-free survival (DFS) in patients diagnosed with well-differentiated papillary thyroid carcinoma who underwent total thyroidectomy.

As a secondary objective, we aimed to identify clinical and histopathological factors associated with structural disease recurrence.

Structural recurrence was defined as the presence of thyroid tissue confirmed by cytology or core needle biopsy during follow-up after total thyroidectomy.

Inclusion criteria were: patients over 18 years of age, treated within the Brazilian public health system (SUS), diagnosed with thyroid nodules classified according to the Bethesda System for Reporting Thyroid Cytopathology, and who underwent total thyroidectomy at the Dr. Arnaldo Cancer Institute.

In cases with benign cytology (Bethesda II), surgery was indicated for patients with compressive symptoms or aesthetic complaints; malignancy was later confirmed on final histopathology.

The study considered the original cytological result without performing slide review or repeat fine-needle aspiration at our institution.

For cases with unsatisfactory or indeterminate cytology (Bethesda I or III), molecular testing was not performed due to unavailability in the public health system. Therefore, only patients with two Bethesda I/III results within a 6-month interval were included.

All histopathological assessments were conducted using standardized protocols, identical equipment, and interpreted by the same dedicated pathology team throughout the study period, ensuring consistency of diagnostic criteria.

Patients with histopathological diagnosis of medullary, anaplastic, or other poorly differentiated thyroid carcinomas were excluded, as were those with a history of hyperthyroidism or prior thyroidectomy performed at external institutions.

For data processing, analysis, and visualization, we used Python (version 3.12.1) with the pandas, numpy, matplotlib, and seaborn libraries.

Statistical analyses included Pearson correlation, chi-square test, and logistic regression to assess associations between categorical variables and the binary target variable. Linear regression was also used to explore relationships between categorical variables in multivariate analysis. A p-value <0.05 was considered statistically significant.

## Results

Between 12 April 2014, and 24 November 2024, a total of 750 patients underwent total thyroidectomy for histologically confirmed well-differentiated papillary thyroid carcinoma (Table 1). The duration of postoperative follow-up ranged between 6 and 100 months, with a maximum follow-up period of 100 months.

**TABLE 1:** Clinical and histopathological categorical variables.

Variable	Category	Absolute frequency	Relative frequency (%)
Gender	Female	457	60.9
Gender	Male	293	39.1
Age	17–29	50	6.7
Age	30–39	143	19.1
Age	40–49	191	25.5
Age	50–59	191	25.5
Age	≥60	175	23.3
Smoking	No	648	86.4
Smoking	Yes	102	13.6
Alcoholism	No	711	94.8
Alcoholism	Yes	39	5.2
FNA (Bethesda)	I	9	1.2
FNA (Bethesda)	II	34	4.5
FNA (Bethesda)	III	145	19.3
FNA (Bethesda)	IV	94	12.5
FNA (Bethesda)	V	255	34
FNA (Bethesda)	VI	213	28.4
pT staging	1	488	65.1
pT staging	2	138	18.4
pT staging	3	124	16.5
pN staging	0	631	84.1
pN staging	1a	74	9.9
pN staging	1b	45	6
Extrathyroidal Extension	Absent	653	87.1
Extrathyroidal Extension	Present	97	12.9
Perineural Invasion	No	704	93.9
Perineural Invasion	Yes	46	6.1
Vascular Invasion	No	674	89.9
Vascular Invasion	Yes	76	10.1
Radioiodine Therapy	No	549	73.2
Radioiodine Therapy	Yes	201	26.8

Of the total cohort, 457 patients (60.93%) were female. The mean age at the time of surgery was 49.14 years (SD = 13.14). Active tobacco use was reported by 102 individuals (13.6%), while 39 patients (5.2%) reported regular alcohol consumption.

**FIGURE 1:**
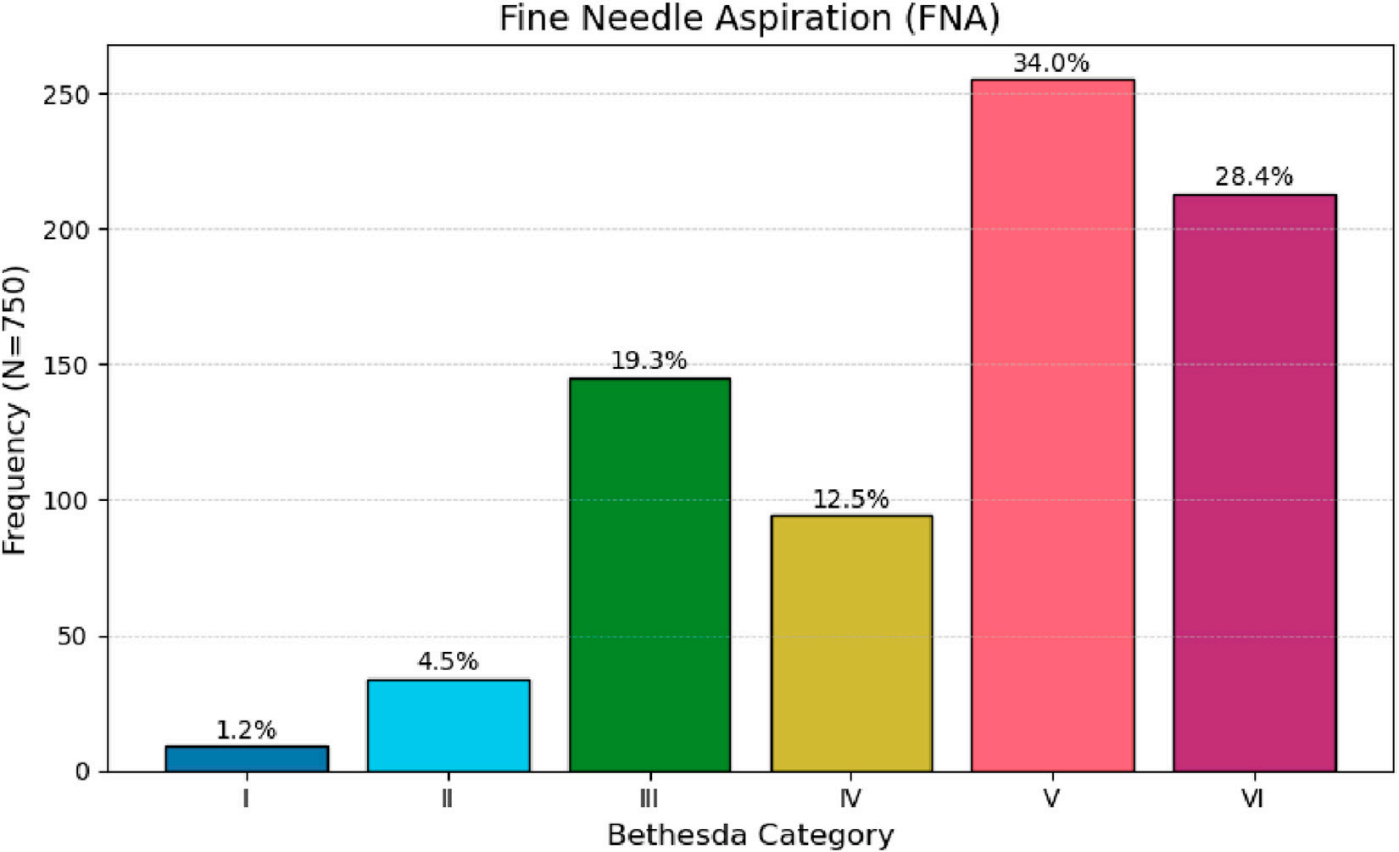
Distribution of Bethesda System categories for thyroid cytopathology in 750 patients.

Preoperative fine-needle aspiration cytology (FNA) of the thyroid nodule yielded suspicious or malignant cytological findings in 62.4% of cases (Table 1; Figure 1).

Ultrasound-based TIRADS classification was documented in only 235 patients (31.3%) and was therefore not incorporated into the exploratory analyses.

**FIGURE 2:**
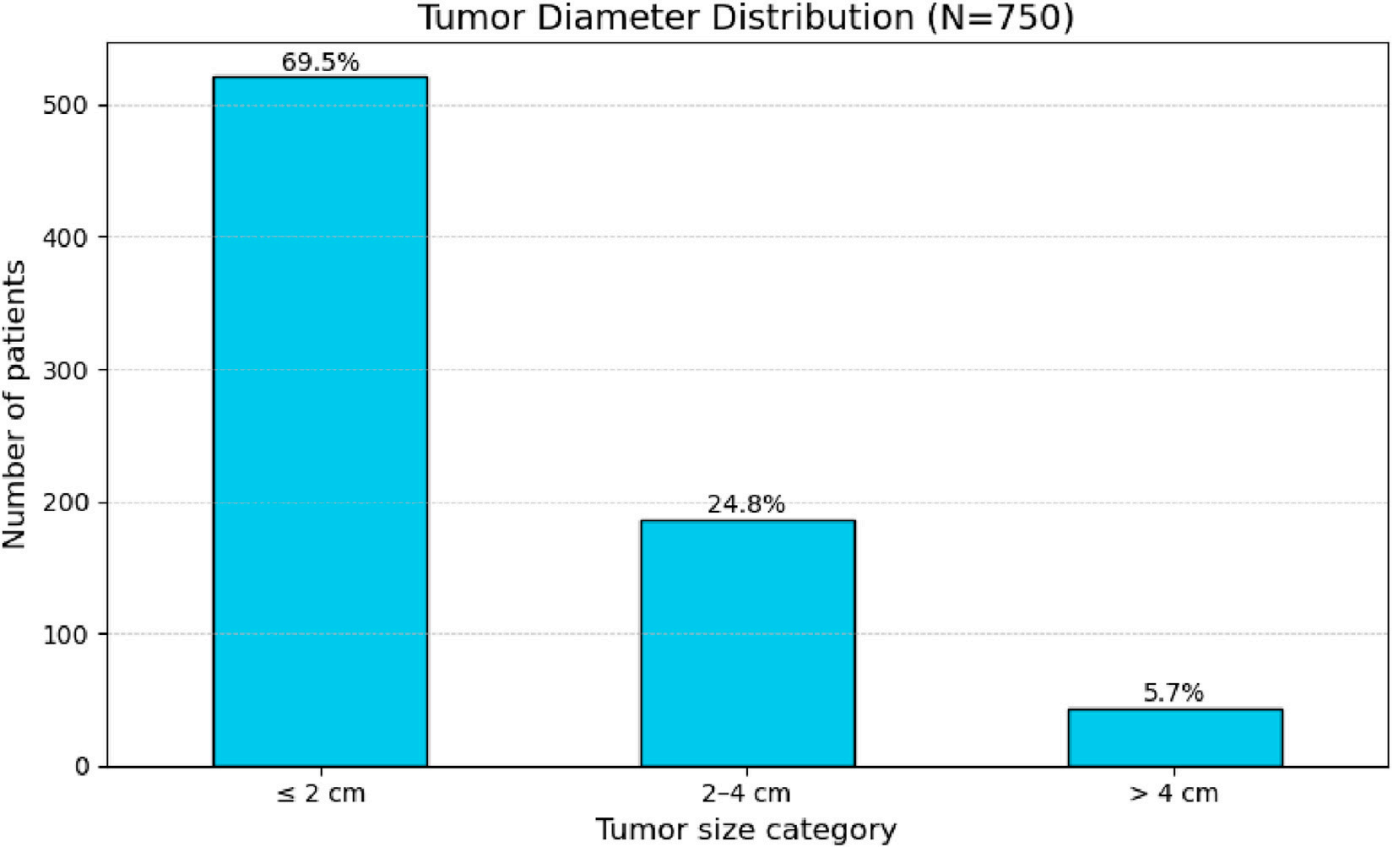
Frequency distribution of tumor size categories (diameter in cm) in 750 patients.

Tumor diameter ranged from 0.2 cm to 11.0 cm, with a mean of 1.8 cm and a standard deviation of 1.4 cm (Table 2). When categorized by size, the majority of nodules (69.5%) measured up to 2 cm, followed by 24.8% measuring between 2 and 4 cm, and 5.7% larger than 4 cm (Figure 2).

**TABLE 2:** Descriptive statistics for continuous histopathological variables.

Variable	Mean	SD	Min	P25	P75	Max
Resected Lymph Nodes	3.3	8.2	0	0	3	74
Metastatic Lymph Nodes	2.5	5.8	0	0	2	51
Tumor Diameter (cm)	1.8	1.4	0.2	0.9	2.4	11

Histopathological characteristics of the surgical specimens were assessed. All selected patients were diagnosed with papillary thyroid carcinoma. According to the eighth edition of the AJCC TNM classification, 488 patients (65.1%) were classified as pT1. The distribution for pT2 and pT3 was 18.4% and 16.5%, respectively.

Central compartment neck dissection was performed in 233 patients, while lateral neck dissection was conducted in 45 cases. A total of 472 patients did not undergo any cervical lymph node dissection. Furthermore, among the entire cohort, 84.1% of patients showed no evidence of lymph node metastasis.

Extrathyroidal extension was absent in 653 patients (87.1%) and present in 97 cases (12.9%). Perineural invasion was identified in 33 patients (5.9%), and vascular invasion was observed in 68 cases (12.2%).

**FIGURE 3:**
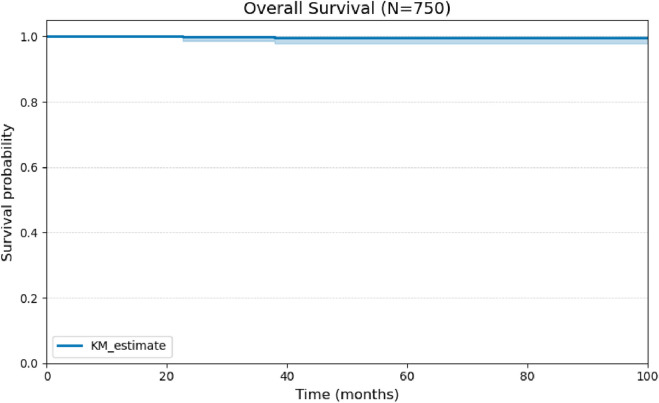
Kaplan-Meier curve for Overall Survival (OS) of the cohort of 750 patients over 100 months of follow-up.

Overall survival at 100 months was 99% (Figure 3). The relationship between histopathological variables and disease-free survival was analyzed. During the follow-up period, 30 cases of structural recurrence were recorded, corresponding to 4% of the cohort. Death occurred in only two patients.

**FIGURE 4:**
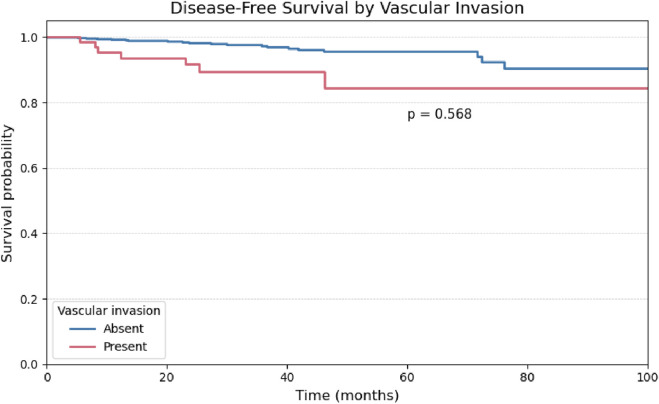
Kaplan-Meier curve for Disease-Free Survival stratified by the presence or absence of Extrathyroidal Extension in 750 patients.

**FIGURE 5:**
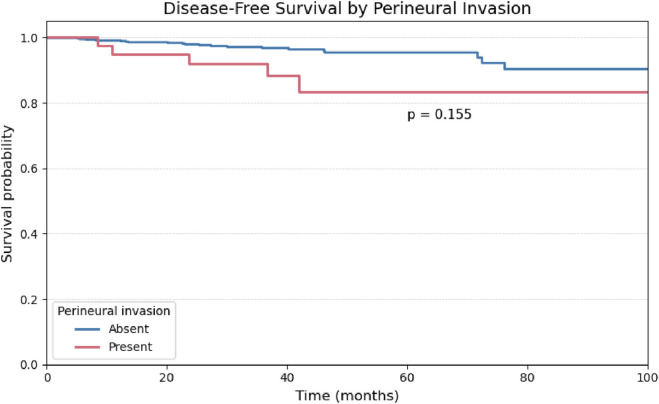
Kaplan-Meier curve for Disease-Free Survival stratified by the presence or absence of Vascular Invasion.

A univariate analysis was performed for the remaining binary histopathological variables. The presence of vascular and perineural invasion demonstrated comparable behavior, with no statistically significant impact on disease-free survival (Figures 4, 5).

**FIGURE 6:**
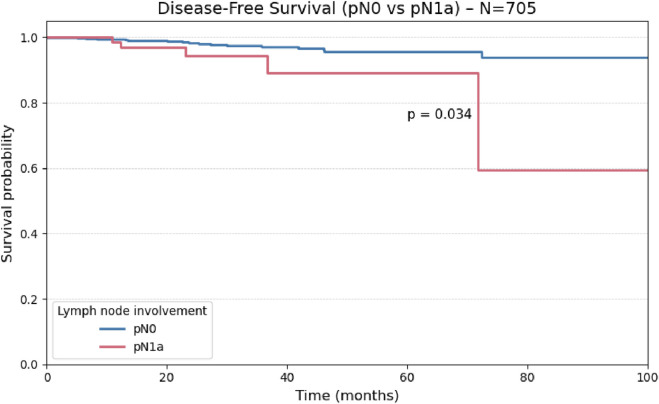
Kaplan-Meier curve for Disease-Free Survival stratified by the presence or absence of Perineural Invasion.

Considering patients classified as pN0 or pN1a (n = 705), central compartment lymph node involvement was significantly associated with reduced disease-free survival (p = 0.03), as illustrated in Figure 6.

**FIGURE 7:**
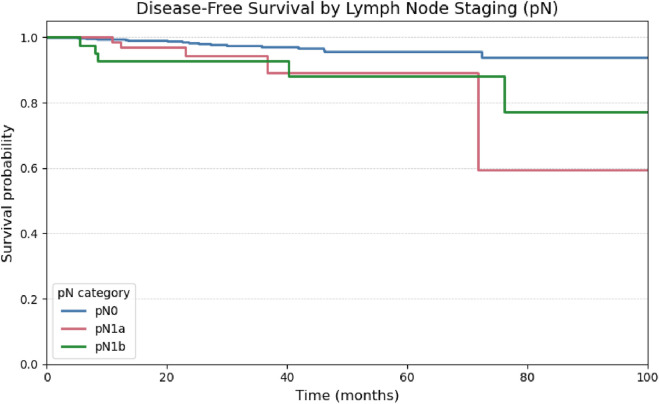
Kaplan-Meier curve for Disease-Free Survival comparing patients with central lymph node involvement (pN1a) versus absence of lymph node involvement (pN0) (N = 705).

Comparisons using the log-rank test demonstrated a statistically significant reduction in disease-free survival among patients with central (pN1a; p = 0.034) and lateral (pN1b; p = 0.010) lymph node metastases when compared to node-negative patients (pN0), indicating a negative prognostic impact of nodal involvement. However, no significant difference in disease-free survival was observed between the pN1a and pN1b subgroups (p = 0.982), as illustrated in Figure 7.

Regarding tumor size based on histopathological evaluation, the primary tumor diameter was stratified into three categories: ≤2 cm (n = 501), 2.1–4.0 cm (n = 164), and >4.0 cm (n = 40). Patients with tumors ≤2 cm demonstrated superior disease-free survival compared to the other groups. Statistically significant differences in survival outcomes were observed among these subgroups (global log-rank test, χ^2^ = 10.58; p = 0.01), as illustrated in Figure 7.

Based on survival analysis (Figure 7), patients with tumors larger than 4 cm demonstrated a significantly increased risk of structural recurrence compared to those with tumors measuring 2 cm or less (p = 0.001). The comparison between tumors ≤2 cm and those measuring between 2.1 and 4.0 cm showed a trend toward statistical significance (p = 0.05), whereas no significant difference was observed between the 2.1–4.0 cm and >4.0 cm groups (p = 0.13).

Due to substantial missingness in RAI dose and timing, RAI was not incorporated into the primary multivariable models. In an unadjusted exploratory Cox analysis comparing patients who ever received RAI (n = 201) *versus* those who never did (n = 549), the hazard of structural recurrence was higher among RAI recipients (HR 6.79, 95% CI 2.89–15.97; *p* < 0.005; concordance 0.74). This estimate is likely influenced by confounding by indication and potential exposure misclassification over follow-up; therefore, it should not be interpreted as causal. These data are provided for completeness and transparency.

In the multivariate Cox regression model (Table 3), only tumor diameter and extrathyroidal extension remained independently associated with structural recurrence. Tumor diameter continued to demonstrate a statistically significant association (HR: 1.30; 95% CI: 1.04–1.63; p = 0.019), even as extrathyroidal extension (HR: 3.21; 95% CI: 1.32–7.77; p = 0.010).

**TABLE 3:** Univariate and multivariate analysis of disease-free survival. Hazard ratios (HR) with 95% confidence intervals (95% CI) were calculated using a Cox regression model.

Variable	HR (95% CI) univariate	p Univariate	HR (95% CI) multivariate	p Multivariate
Age	0.97 (0.95–1.00)	0.047	0.98 (0.95–1.00)	0.074
Tumor diameter (cm)	1.37 (1.14–1.61)	<0.001	1.30 (1.04–1.63)	0.019
Extrathyroidal extension	4.94 (2.36–10.34)	<0.001	3.21 (1.32–7.77)	0.01
Perineural invasion	3.13 (1.26–7.76)	0.014	0.91 (0.30–2.71)	0.858
Vascular invasion	2.85 (1.34–6.06)	0.007	1.32 (0.50–3.49)	0.571
pN1a	2.43 (0.92–6.41)	0.074	1.31 (0.42–4.04)	0.639
pN1b	2.86 (1.12–7.27)	0.027	1.88 (0.64–5.52)	0.253

Perineural invasion (HR: 0.91; 95% CI: 0.30–2.71; p = 0.858), vascular invasion (HR: 1.32; 95% CI: 0.50–3.49; p = 0.571), and lateral lymph node metastasis (pN1b) (HR: 1.88; 95% CI: 0.64–5.52; p = 0.253) did not retain statistical significance in the adjusted model.

Age demonstrated a marginal inverse association with structural recurrence (HR: 0.97; 95% CI: 0.95–1.00; p = 0.047), suggesting a potential protective effect in older patients. Central lymph node metastasis (pN1a) did not reach statistical significance in the univariate model (HR: 2.43; 95% CI: 0.92–6.41; p = 0.074).

After adjustment in the multivariable Cox proportional hazards model, only tumor diameter (HR: 1.30; 95% CI: 1.04–1.63; p = 0.019) and extrathyroidal extension (HR: 3.21; 95% CI: 1.32–7.77; p = 0.010) remained as independent predictors of structural recurrence.

The prognostic significance observed in univariate analysis for other variables did not persist in the adjusted model, including perineural invasion (HR: 0.91; 95% CI: 0.30–2.71; p = 0.858), vascular invasion (HR: 1.32; 95% CI: 0.50–3.49; p = 0.571), central compartment lymph node metastasis (pN1a; HR: 1.31; 95% CI: 0.42–4.04; p = 0.639), and lateral cervical lymph node involvement (pN1b; HR: 1.88; 95% CI: 0.64–5.52; p = 0.253). While some of these variables exhibited a numerical trend toward increased risk, they did not reach statistical significance in the multivariate analysis.

## Discussion

In this retrospective cohort study, we identified the main clinical and histopathological factors associated with structural recurrence in patients with well-differentiated papillary thyroid carcinoma. The structural recurrence rate was 4% (30 cases), characterizing a lowfrequency event, in line with international data. Medas et al. reported a 5-year structural recurrence rate of 6.2% in a retrospective series of 579 cases of differentiated thyroid carcinoma (4). Similarly, Iizuka et al. observed a recurrence rate of 13.7% over a 3-year follow-up among 284 intermediate- and highrisk patients after radioiodine therapy (3).

All patients in our study underwent surgery performed by a specialized team at a high-volume academic cancer center, which may have contributed to the lower recurrence rates observed. This hypothesis is supported by findings from Loyo et al., who, in a large cohort of 871,644 patients undergoing thyroidectomy, reported improved short and long term outcomes in high volume surgical centers (9).

**FIGURE 8:**
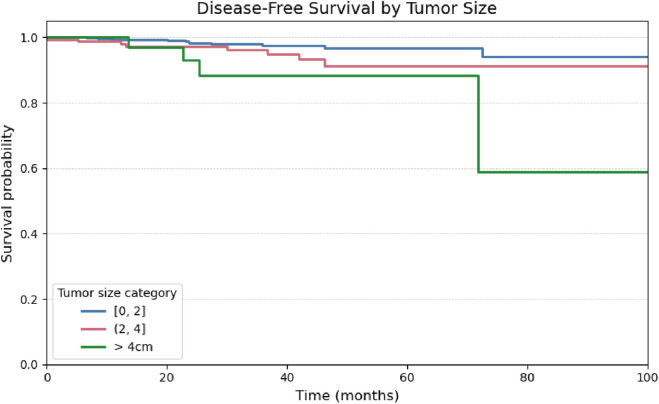
Kaplan-Meier curve for Disease-Free Survival stratified by pathological lymph node staging (pN0, pN1a, pN1b).

Tumor diameter was a significant prognostic factor for structural recurrence in both univariate and multivariate analyses (Figure 8; Table 3). This result is consistent with recent studies, including those by Can et al. (10) and Kurtom et al. (6), which reinforce the favorable prognosis associated with papillary thyroid microcarcinomas.

**FIGURE 9:**
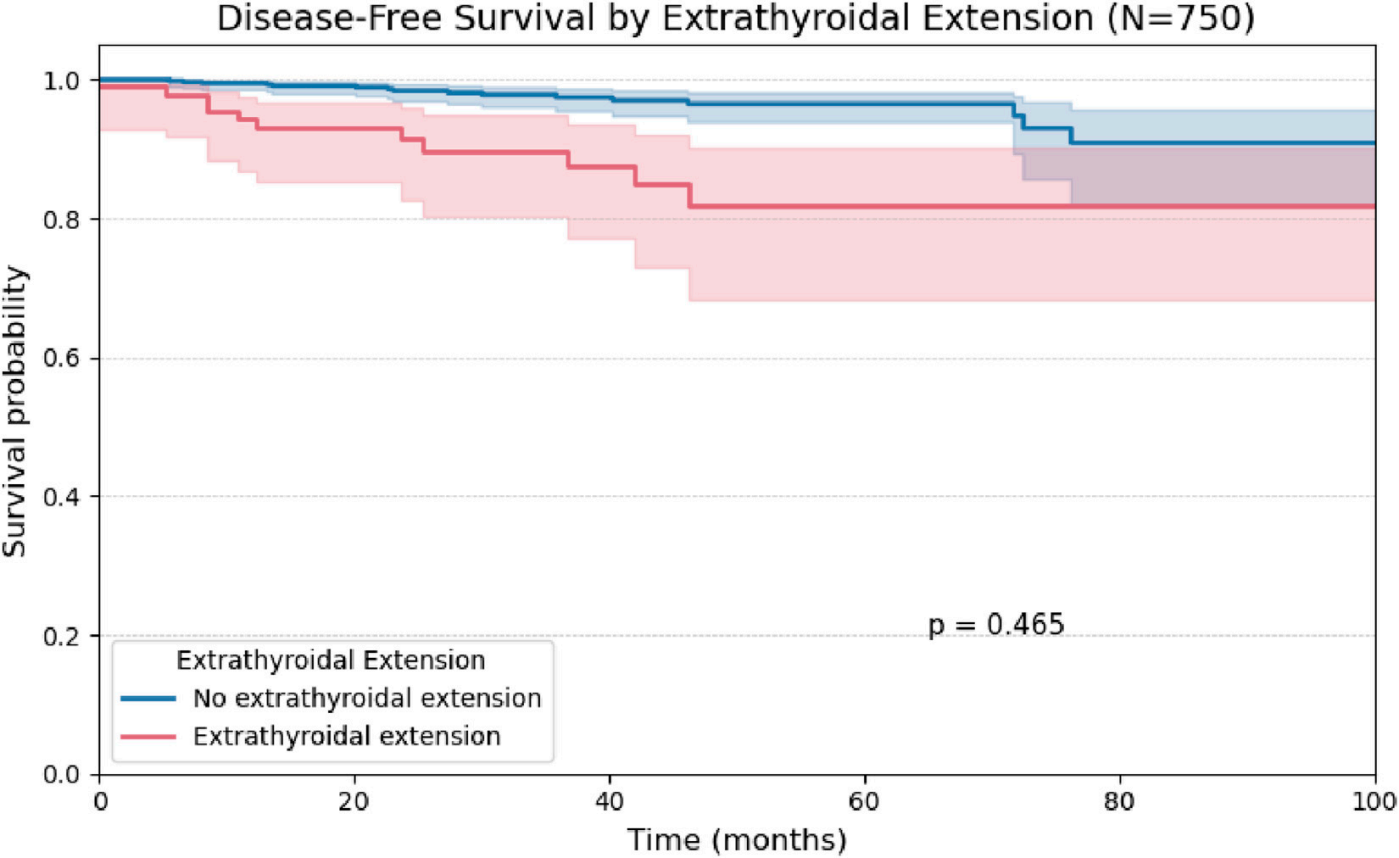
Kaplan-Meier curve for Disease-Free Survival stratified by tumor size categories (≤ 2 cm, >2 to 4 cm, > 4 cm).

Although Kaplan-Meier analysis using the log-rank test did not demonstrate a statistically significant difference in disease-free survival based on the presence of extrathyroidal extension (p = 0.40; Figure 9), this variable remained the strongest predictor of recurrence in Cox regression analysis, with a hazard ratio of 4.94 in the univariate model and 3.21 in the multivariate model (Table 4). This discrepancy may be explained by methodological differences between the tests: while the log-rank test evaluates global survival curve differences, the Cox model estimates the continuous effect of each covariate on recurrence risk over time, and is often more sensitive in analyses involving low event rates (6).

The low incidence of structural recurrence (30/750; 4.0%) implies—by Schoenfeld’s event-driven method (extrathyroidal extension prevalence 12.9%, target HR = 1.70, two-sided α = 0.05, 80% power)—a requirement of about 248 events (approximately 6,200 patients assuming a 4% event fraction). With only 30 events, the power to detect HR = 1.70 is approximately 16%, which justifies parsimonious modeling and an emphasis on effect sizes and confidence intervals rather than statistical significance alone.

The prognostic relevance of extrathyroidal extension supports its inclusion as a high-risk criterion in the 2015 American Thyroid Association guidelines, and its role as an indication for adjuvant radioactive iodine therapy (5,11). Anwar et al. (2022), using a similar methodology in a smaller cohort (n = 312), also identified extrathyroidal extension as the primary independent risk factor for recurrence (12). Consistent findings were reported by Weis et al. in a population-based analysis of 101,087 cases from the SEER (Surveillance, Epidemiology, and End Results) database (13). However, perineural invasion, lymphovascular invasion, and central lymph node metastasis, as well as age did not significantly impact disease-free survival in either univariate or multivariate models, corroborating previous literature. In agreement with our findings, a Japanese cohort described by Ito et al. (2012) concluded that age is not an independent predictor of recurrence, although it is associated with higher overall mortality in advanced-stage disease (14).

Similarly, Xing et al., in a retrospective analysis of 1,849 patients, found that neither central lymph node involvement nor vascular invasion were associated with recurrence risk in multivariate models, particularly in tumors smaller than 4 cm (15). Although the study by Xing incorporated the BRAF V600E mutation in its prognostic analysis alongside clinicopathologic features, molecular testing is not yet routinely available in the Brazilian public health system for pre- or postoperative risk stratification (15).

While some variables may be associated with prognosis in selected subgroups, it is important to acknowledge that statistical models for survival and recurrence reflect the behavior of relatively homogeneous populations. Therefore, it is unlikely that a single model can accurately predict structural recurrence across heterogeneous clinical settings.

Given Brazil’s ethnic, cultural, and socioeconomic diversity, there is an opportunity to develop robust, regionally adapted predictive models to identify patients who would benefit most from adjuvant therapy or prolonged surveillance. Expansion of this study to other high-volume referral centers for thyroid cancer treatment across the country is thus warranted to enhance the generalizability of these findings.

## Conclusion

In this retrospective study involving 750 patients who underwent total thyroidectomy for well-differentiated papillary thyroid carcinoma, structural recurrence was identified as a rare event, with a recurrence rate of 4% even over extended follow-up. The main factors independently associated with increased risk of structural recurrence were extrathyroidal extension and tumor diameter greater than 2 cm, both of which remained statistically significant in univariate and multivariate analyses, in accordance with international literature.

Clinical variables such as age, perineural invasion, and lymphovascular invasion did not demonstrate significant impact on recurrence-free survival.

We recommend the development of multicenter studies to validate these findings across other high-volume reference centers in Brazil and to support the construction of regionally adapted statistical models for recurrence risk stratification.

## Data Availability

The raw data supporting the conclusions of this article will be made available by the authors, without undue reservation.
